# New perspectives on chemokines in hepatocellular carcinoma therapy: a critical pathway for natural products regulation of the tumor microenvironment

**DOI:** 10.3389/fimmu.2024.1456405

**Published:** 2024-08-14

**Authors:** Xie Ruishi, Xu Linyi, Bai Yunfan, Yu Wenbo, Zhang Xiaoying, Fang Xiaoxue, Zhu Difu, Lan Xintian, Zhu Ming, Luo Haoming

**Affiliations:** ^1^ School of Pharmacy, Changchun University of Chinese Medicine, Changchun, Jilin, China; ^2^ The First Hospital of Jilin University, Changchun, China

**Keywords:** chemokines, natural products, hepatocellular carcinoma, tumor microenvironment, immune intervention

## Abstract

Hepatocellular carcinoma (HCC) is one of the most common primary neoplasms of the liver and one of the most common solid tumors in the world. Its global incidence is increasing and it has become the third leading cause of cancer-related deaths. There is growing evidence that chemokines play an important role in the tumor microenvironment, regulating the migration and localization of immune cells in tissues and are critical for the function of the immune system. This review comprehensively analyses the expression and activity of chemokines in the TME of HCC and describes their interrelationship with hepatocarcinogenesis and progression. Special attention is given to the role of chemokine-chemokine receptors in the regulation of immune cell accumulation in the TME. Therapeutic strategies targeting tumor-promoting chemokines or the induction/release of beneficial chemokines are reviewed, highlighting the potential value of natural products in modulating chemokines and their receptors in the treatment of HCC. The in-depth discussion in this paper provides a theoretical basis for the treatment of HCC. It is an important reference for new drug development and clinical research.

## 1 Introduction

Hepatocellular carcinoma (HCC) is one of the most common primary neoplasms of the liver and one of the most common solid tumors in the world (1). More so than other cancers, chronic inflammation is a hallmark of HCC, with 90% of diagnoses occurring in the context of chronic liver disease (2). The incidence of HCC is on the rise worldwide and has become the third most common cause of cancer-related deaths (3). The main risk factors are infection with the hepatitis B virus (HBV) or the hepatitis C virus (HCV) (4), metabolic-associated fatty liver disease (MAFLD) (5), smoking (6), obesity and type 2 diabetes mellitus(T2DM) (7), etc. Inflammation is a major contributor to the pathogenesis of HCC (8).

The tumor microenvironment (TME) is a key determinant of tumor growth and consists of tumor cells, numerous immune cells, vascular and stromal cells, etc (9). Chemokines, vital cytokines capable of altering the TME, constitute the largest subset of cell factors (10). During inflammatory responses, chemokines are regarded as primary drivers for immune cell infiltration into the liver, including macrophages, neutrophils, and other cells (11). To date, more than 50 chemokines have been identified in humans and mice. Chemokines are categorized into families according to the structural motifs of their N-terminal cysteines: C-C motif chemokine ligands (CC), C-X-C motif chemokine ligands (CXC), X-C motif chemokine ligands (XC), and CX3C motif chemokine ligands (CX3C) (12), where C represents cysteine and X denotes any amino acid residue (13). These chemotactic factors signal through seven-transmembrane chemokine receptors coupled with cell surface G-proteins, thereby stimulating directed cell migration (14). Chemokine receptors are classified into four subfamilies based on their binding to different ligands: CC Chemokine Receptors (CCR), CXC chemokine receptors (CXCR), XC chemokine receptors (XCR), and CX3C chemokine receptors (CX3CR) (15). Constitutive chemokines are expressed under physiological conditions and play roles in cell migration and homing (16), while inflammatory chemokines are rapidly secreted at sites of inflammation to recruit effector cells to inflamed tissues (17). In TME, tumor cells and immune cells express multiple chemokine receptors on their surfaces. This diversity leads to differential responses to chemokines, which in turn affects their migration and function, regulating the tumor immune response, as well as tumor progression and prognosis, significantly influencing tumor therapy (18). A substantial body of research substantiates that CC chemokines (e.g., CCL2, CCL5) and CXC chemokines (e.g., CXCL1, CXCL2, CXCL5) recruit diverse immune cells, such as CCR2+ monocytes and CXCR2+ neutrophils, to tumor sites. These cells subsequently differentiate into tumor-associated macrophages (TAMs) and tumor-associated neutrophils (TANs), thereby exerting either promotive or inhibitory effects on tumorigenesis (19, 20). For instance, studies in HCC have demonstrated that the deletion of CCL5 enhances CXCL1 expression in neutrophils. This upregulation activates the CXCL1-CXCR2 axis, promoting neutrophil infiltration into the liver and consequently exacerbating inflammatory liver damage (21). CCL15 recruits CCR1+CD14+ monocytes to the invasive front of HCC, thereby suppressing antitumor immune responses, promoting angiogenesis, and accelerating the metastasis of HCC cells. Notably, blockade of the CCL15-CCR1 axis has been shown to reduce the proliferation and migration of HCC cells *in vivo (* 22). In contrast, Brandt et al. elucidated that the chemokine receptor CXCR3 orchestrates the polarization of TAMs, effectively constraining tumor growth and angiogenesis in murine hepatocellular carcinoma (HCC) (23). In conclusion, chemokines and their receptors are promising biomarkers and therapeutic targets for the diagnosis and immunotherapy of hepatocellular carcinoma, and an in-depth understanding of the regulatory mechanisms of chemokines is essential for the development of novel therapeutic strategies (24).

Currently, natural products has been demonstrated to play a significant role in the prevention of HCC, as well as in the control of its metastasis and recurrence (25). These natural products modulate chemokine expression and function, as well as the interaction between chemokines and their receptors, to influence the immune system and microenvironment in the body, thereby suppressing the progression of HCC (26). Reportedly, the natural product 747 from Abies georgei exhibits sensitivity and selectivity as a CCR2 antagonist in HCC mouse models. It effectively blocks tumor-infiltrating macrophage-mediated immune suppression, increases the number of CD8+ T cells within tumors, and suppresses the growth of both *in situ* and subcutaneous tumors in a CD8+ T cell-dependent manner (27). Triptolide, derived from Tripterygium wilfordii Hook.f. extract, is a monomeric compound renowned for its diverse pharmacological properties. It functions as an inhibitor of the miR-532-5p/CXCL2 axis, effectively suppressing the migration of HCC cells and inhibiting the initiation and metastasis of HCC tumors (28). Aconite alkaloid extract (AAE) induces the upregulation of CCL2 via activation of c-Jun N-terminal kinase (JNK), thereby enhancing natural killer (NK) cell infiltration to suppress the growth of HCC (29). These findings suggest that leveraging natural products to modulate chemokine actions and adjust the tumor microenvironment composition can bolster immune responses in patients, presenting a promising and clinically valuable therapeutic strategy for HCC (30). This article provides a comprehensive review of the effects of metabolites derived from natural products on the regulation of chemokines and their receptors in the HCC tumor microenvironment, revealing how natural products regulate chemokines through specific molecular pathways, thereby affecting the tumor microenvironment and the development of HCC. We summarizes the research progress of plant metabolites in the treatment of hepatocellular carcinoma by affecting chemokines and discusses their potential developmental trends and shortcomings.

## 2 Tumor microenvironment and chemokines

TME constitutes a complex ecosystem comprising tumor cells, immune cells, stromal cells, and vascular networks. Beyond supporting tumor cell growth, the TME plays a pivotal role in tumor immune evasion and drug resistance (10). TME exhibits dynamic changes, wherein the distribution and functional status of immune cells evolve continuously with tumor progression (31). Chemokines are a class of cytokines that direct immune cell migration by binding to specific receptors. Within the TME, chemokines not only govern the recruitment and directional migration of immune cells but also impact their activation states, thus playing pivotal roles in tumor immune surveillance and evasion (32).

### 2.1 Chemokines and tumor-associated macrophages

TAMs constitute a critical component of the immune microenvironment in HCC (33), primarily recruited and polarized into an M2 phenotype by various cytokines (34, 35). It is widely accepted that TAMs originate from peripheral blood monocytes (PBMCs) and are recruited to the tumor vicinity by chemokines secreted within the tumor microenvironment, such as CCL2. Once within the tumor microenvironment, monocytes undergo polarization into M2-like macrophages (36). These macrophages secrete anti-inflammatory cytokines, promote angiogenesis, exert anti-inflammatory effects, and contribute to stromal remodeling. This polarization process leads to apoptosis of CD8+ T lymphocytes, suppression of Th1-type immune responses, reshaping of the microenvironment, alteration of immune homeostasis, and ultimately facilitates tumor growth, invasion, and metastasis (37). Concurrently, TAMs stimulate the secretion of CXCL1, promoting the polarization of M2 macrophages and thereby influencing the migration and invasion of HCC cells (38). Targeting the CCL2/CCR2 axis and CXCL1/CXCR2 blockade therapy can inhibit the recruitment and M2 polarization of inflammatory monocytes/infiltrative TAMs, disrupting immune suppression within the TME (39). Consequently, reshaping the immunosuppressive TME and repolarizing TAMs from an M2 to an anti-tumor phenotype emerges as a promising cancer immunotherapy strategy. This approach can activate anti-tumor immune responses, enhance the efficacy of cancer immunotherapy, and thereby restrict tumor progression (40).

### 2.2 Chemokines and tumor-associated neutrophils

Within the TME, neutrophils undergo moderate activation to become TANs, releasing reactive oxygen species (ROS), neutrophil elastase (NE), and other bioactive substances that promote tumor growth and invasion (41). TANs display two distinct phenotypes categorized as the anti-tumor N1 and pro-tumor N2 phenotypes (42, 43). Research has revealed that TANs enhance stem cell-like characteristics in HCC through activation of the miR-301b-3p/LSAMP/CYLD pathway. These stem cell-like HCC cells secrete the chemokine CXCL5, which in turn recruits intratumoral TANs, thereby facilitating the invasion and metastasis of HCC (44). Xu et al. explored the role of CCL21 in the TME of HCC using *in vitro* and *in vivo* HCC subcutaneous tumor models with neutrophils. Their findings indicate that CCL21 inhibits the polarization of N2 neutrophils by suppressing the activation of the nuclear factor κB (NF-κB) pathway (45). In the future, targeting the recruitment, migration, or activation of neutrophils may offer promising avenues for anti-tumor therapy.

### 2.3 Chemokines and myeloid-derived suppressor cells

Myeloid-derived suppressor cells (MDSCs) are highly heterogeneous suppressive immune cells distributed across the bone marrow, spleen, peripheral blood, and tumor tissues (46). MDSCs can be categorized into two subsets: monocytic MDSCs (M-MDSCs) and polymorphonuclear or granulocytic MDSCs (PMN-MDSCs) (47). In HCC, reduced RIP3 expression has been observed in patients, validating its capacity to upregulate CXCL1 expression within HCC cells. This upregulation promotes the recruitment of MDSCs through the CXCL1-CXCR2 axis, thereby fostering the progression of HCC. Targeting the CXCL1-CXCR2 chemotactic pathway presents a potential immunotherapeutic strategy to impede the advancement of RIP3-deficient HCC (48).In H22 orthotopic HCC mice, PMN-MDSCs exhibit increased prevalence compared to M-MDSCs, predominating in the spleen, peripheral blood, and tumor tissue. CCL9 and CCL2 play a role in mobilizing PMN-MDSCs from the spleen to peripheral blood, thereby facilitating tumor initiation and growth (49). Hence, therapeutic strategies aimed at modulating MDSCs accumulation and activation hold promise as treatments for malignant liver diseases.

### 2.4 Chemokines and cancer-associated fibroblasts

One of the hallmark features of HCC is liver fibrosis, observed in approximately 80% to 90% of clinical cases (50). The development of fibrosis often involves the infiltration of immune cells and stromal cells, including hepatic stellate cells (HSCs), a significant source of cancer-associated fibroblasts (CAFs) (51). Luo et al. demonstrated that CAFs promote HCC stem-like characteristics by inducing the expression of Forkhead box Q1 (FOXQ1) in HCC cells and activating N-myc downstream-regulated gene 1 (NDRG1). Furthermore, activated HCC cells can secrete chemokine ligand 26 (CCL26) to recruit additional CAFs, thereby driving HCC progression (52). In HCC, CAFs secrete higher levels of chemokines, including CCL2, CCL5, CCL7, and CXCL16, compared to neighboring fibroblasts. These chemokines play pivotal roles in recruiting immune cells, thus facilitating HCC metastasis and invasion (53). This body of evidence underscores the involvement of CAFs in immune suppression through the modulation of interactions among diverse immune cell types via chemokine signaling.

### 2.5 Chemokines and tumor-infiltrating lymphocytes

Tumor-infiltrating lymphocytes (TILs) are a pivotal component of the HCC microenvironment, encompassing regulatory T cells (Tregs), NK cells, cytotoxic T lymphocytes (CTLs), and B cells (54). Tregs are a subset of immune cells known for their potent immunosuppressive effects (55). Studies have reported a significant increase in CD4+ CD25+ Tregs in the peritumoral regions of HCC, with this increase being correlated with tumor size (56). TAMs secrete the chemokine CCL22, which recruits T cells to the tumor site, thereby enhancing the accumulation of Tregs and fostering an immunosuppressive microenvironment that impedes cytotoxic T cell activation (57). NK cells mediate innate immune responses and possess the ability to directly exert cytotoxic effects without prior sensitization. However, their functionality is often compromised within the tumor microenvironment (58).Recent studies have elucidated that miR-561-5p, which is upregulated in HCC, directly targets and downregulates CX3CL1 expression, thereby inhibiting the chemotactic migration of CX3CR1+ NK cells, promoting tumor cell survival, and facilitating pulmonary metastasis (59).

In conclusion, chemokines play a pivotal role in the TME, orchestrating the recruitment, migration, and activation of immune cells by binding to specific receptors. This regulatory process significantly impacts tumor progression and immune evasion mechanisms. In HCC, chemokines not only drive the polarization and functionality of TAMs, TANs, MDSCs, and CAFs, but also influence the distribution and activity of TILs. Collectively, these roles contribute to the establishment of an immunosuppressive TME that supports tumor growth, invasion, and metastasis.

**Figure 1:**
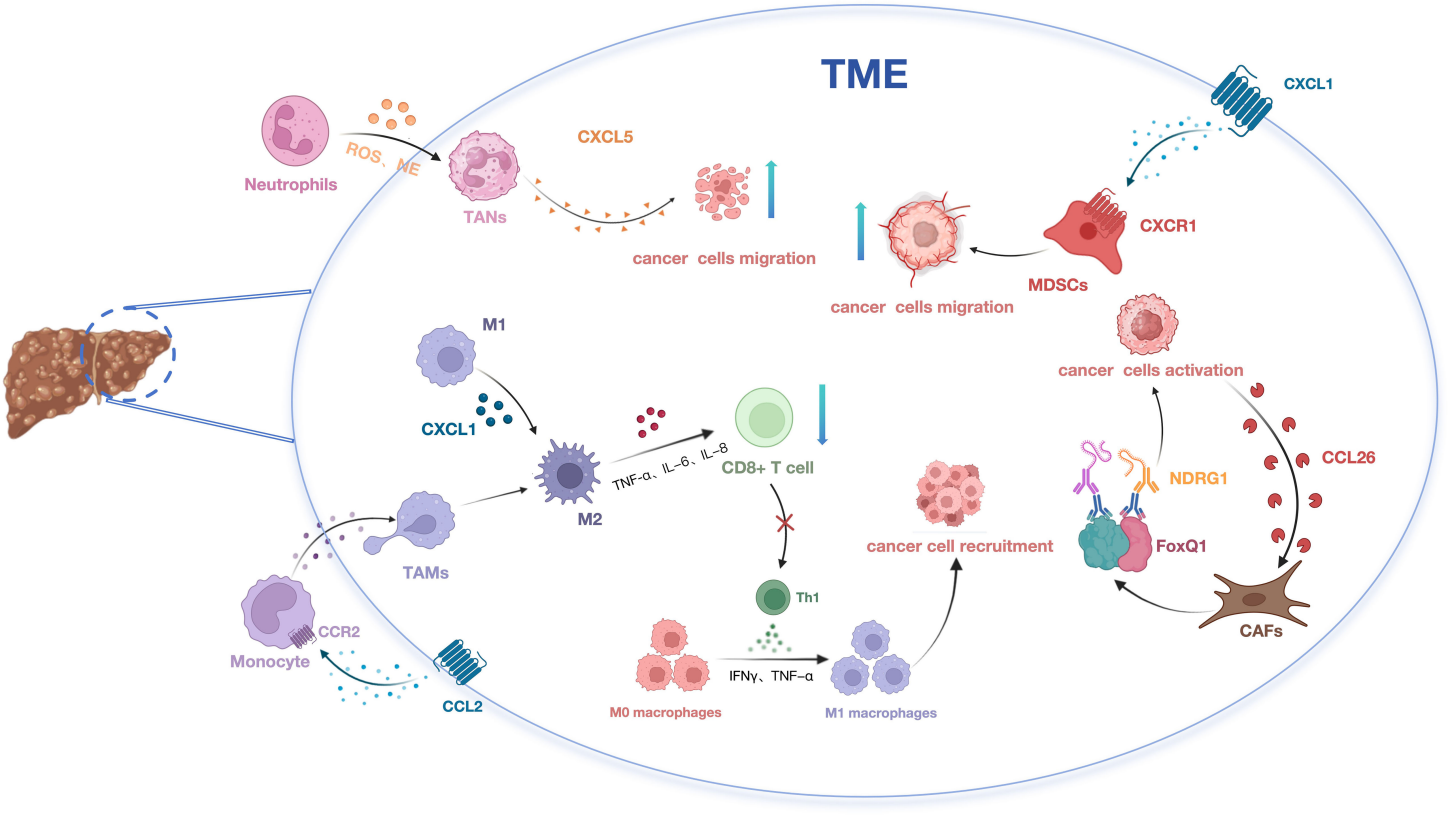
The impact of chemokines on HCC development via modulation of immune cell migration and recruitment.

By modulating chemokines and their associated signaling pathways, the immunosuppressive TME can be reshaped to activate antitumor immune responses. Growing evidence indicates that natural products has unique potential in regulating chemokines and their signaling pathways. Modulating the TME through natural products can effectively inhibit tumor progression. Numerous studies have investigated the mechanisms by which these active components of natural products exert their effects in HCC, thereby advancing the development of immunotherapy for this malignancy (**Figure 1**).

## 3 Role of natural products and their extracts in hepatocellular carcinoma

Natural products are extensively utilized in cancer treatment due to their inherent characteristics. Unlike conventional drugs that target specific symptoms or diseases, natural products often regulate overall bodily functions (60). Compared to chemical drugs, natural products tend to have fewer side effects, which is particularly advantageous for HCC patients with impaired liver function and low drug tolerance. Furthermore, natural products possess unique potential and mechanisms for regulating chemokines to mediate immune cell activity and treat HCC (61). Various natural components, such as phenolics, flavonoids, terpenes, and alkaloids, exhibit notable antitumor activity (**Table 1**).

**Table 1:** Mechanism of natural products and their extracts in the treatment of HCC through the chemokines.

Natural product	Compound Name	Chemokines or chemokine receptors	Machine	References
*Cordyceps militaris*	Cordycepin	CXCR4	Inhibition of p-IκBα activation to suppress P65 nuclear translocation reduces CXCR4 expression, thereby attenuating the migratory and invasive capacities of liver cancer cells	(62)
*Sabia japonica*	Sinomenine	CXCL1/2-CXCR4 CCL21-CCR7	Downregulate CXCL1/2-CXCR4 and CCL21-CCR7 axes expression, while inhibiting the ERK1/2/MMP2/9 signaling pathways	(63)
*Ervatamia microphylla*	Conophylline	CCL2	Inhibit smooth muscle actin expression in cancer-associated fibroblasts (CAFs), and suppress the production of cytokines IL-6, IL-8, CCL2, angiogenin, and osteopontin (OPN) synthesized by CAFs	(64)
*Uncaria rhynchophylla*	Isorhynchophylline	CXCR4	Inhibit Signal Transducer and Activator of Transcription 3 (STAT3) phosphorylation, suppress HepG2 cell migration, and downregulate CXCR4, MMP-9, and MMP-2 expression	(65)
*Tea*	Epigallocatechin-3-gallate, Gallocatechin gallate	CCL2	Epicatechin binds to receptors/GAGs, acting as an inhibitor of CCL2-GPCR interaction and modulating CCL2-mediated leukocyte recruitment	(66)
*Ganoderma resinaceum*	G. resinaceum polysaccharide-rich fractions	CX3CL1/CCL11	Anti-inflammatory and antioxidant properties restrict immune cell infiltration, suppress pro-inflammatory cytokines such as G-CSF, IFNγ, and TNFα, along with eosinophil chemotactic factor and fractalkine expression, while enhancing anti-inflammatory cytokines (IL-10 and IL-12 p70)	(67)
*Cladosiphon okamuranus*	Fucoidan	CXCL12-CXCR4	Binding to CXCL12 results in the downregulation of CXCL12 expression, which induces cell cycle arrest and impedes its function in liver regeneration	(68)
*Saccharina japonica*	Fucosylated glycoproteins (AAL- or LCA-glycoprotein)	CXCR2	Inhibition of migration of human HCC cells through binding to the IL-8 receptor CXCR2	(69)
*Erigeron breviscapus*	Breviscapine	CCL2	Reduced ROS generation results in the inactivation of pro-inflammatory signaling pathways, including TLR4/NF-κB, caspase-3/PARP, and MAPK, leading to decreased secretion of pro-inflammatory cytokines TNF-α, IL-6, IL-1β, and the chemokine MCP-1	(70)
*Epimedium*	Icaritin	CCL2	Induction of caspase-dependent cellular apoptosis, inhibition of hepatocellular carcinoma development via IL-6/Jak2/IL-10 pathways, and reshaping of the immunosuppressive microenvironment using combined doxorubicin nanoparticles. This strategy enhances immune-stimulatory cytokines IFNγ, TNFα, and IL-12, and diminishes immunosuppressive cytokines CCL2, TGFβ, IL-4, IL-6, and IL-10	(71)
*Chloranthus henryi*	Flavokawain A	CXCL12/CXCR 4	Targeting CXCL12 to inhibit CXCR4 activation suppresses HCC cell proliferation, EMT, invasion, metastasis, and vasculogenic mimicry (VM) formation through the PI3K/Akt/NF-κB signaling pathway.	(72)
*Plumbago zeylanica*	Plumbagin	CXCR4/CXCR7	Eliminating SDF-1-induced endothelial tube formation, partially block activation of the angiogenesis signaling pathway and inhibit CXCR4/CXCR7 expression	(73)
*Terminalia bellirica*	Tannin	CCL5/CXCL10	Facilitating TAM polarization from the pro-tumoral M2 phenotype to the immunostimulatory M1 phenotype enhances the immune-suppressive TME; Promoting T cell infiltration mediated by the expression of CCL5 and CXCL10	(74)
*Mylabris phalerata Pallas*	Cantharidin	CXCL10	Factors influencing immune cell trafficking and immune signal responses, potentially regulated by EZH2 in an HDAC10-dependent manner, suppress CXCL10 to inhibit NK cells and contribute to miR-214-regulated macrophage polarization, exerting anti-tumor effects on HCC	(75)
*Tripterygium wilfordii*	Celastrol	CXCR4	In a dose-dependent manner, the expression of CXCR4 and its downstream pathways PI3K and Akt is reduced	(76)
*Bufonis Venenum*	Bufalin	CCR9/CCR10/CXCR4	Inhibit APOBEC3F and intestinal immune network proteins CCR9, CCR10, CXCR4, and pIgR to promote IgA production	(77)
*Schisandra chinensis*	Schisandra chinensis lignans	CCL20	Inhibiting CCL20-induced EMT, invasion, and migration, while enhancing apoptosis in hepatocellular carcinoma cells through the ERK1/2 pathway	(78)
*Cnidium monnieri*	Osthole	CXCL1/CX3CL1	Inhibiting the production of fibrosis- and inflammation-related cytokines and chemokines such as ICAM-1, CD62L, VEGF, CXCL1, and CX3CL1 reduces plasma AST and ALT levels, improves tissue structure, and decreases collagen and α-SMA accumulation, thereby alleviating liver damage	(79)
*Oldenlandia diffusa*	Oldenlandia diffusa active ingredients	CXCR1/CXCR2/CXCR4	Inhibit the migration of hepatocellular carcinoma cells by downregulating migration-related chemokine receptors, such as CXCR1, CXCR2, and CXCR4; induce apoptosis in these cells via the caspase-3 pathway	(80)
*Trichosanthes*	Trichosanthin	CCL2/CCL17/CCL22	Induce caspase-mediated apoptosis in hepatocellular carcinoma cells, upregulate the expression of chemokines CCL2, CCL17, and CCL22, increase the expression of the main receptor for granzyme B in hepatocellular carcinoma tissue, the mannose-6-phosphate receptor (M6PR), and promote recruitment of CD8+ T cells to hepatocellular carcinoma tissue.	(81)
*Aconite*	Aconite aqueous extracts	CCL2	By activating c-Jun N-terminal kinase, CCL2 expression is upregulated to promote NK cell infiltration into tumors	(29)

### 3.1 Alkaloids

Alkaloids, a class of natural compounds extracted from natural products, have been shown to possess significant anti-hepatocellular carcinoma effects (82). Current research indicates that alkaloids exert their anticancer effects by reducing cancer cell migration and invasion through mechanisms such as downregulating the expression of CXCR4, MMP-9, and MMP-2, or inhibiting the CXCL12-CXCR4 and CCL21-CCR7 axes.

#### 3.1.1 Cordycepin

*Cordyceps sinensis* (BerK.) Sacc. exhibits potent anti-hepatocellular carcinoma properties and enhances immune function (83). Key components such as cordycepin, cordyceps polysaccharides, and cordyceps peptides possess anti-inflammatory, antitumor, antiviral, and immunomodulatory effects (84). These components exert anticancer effects by interfering with cancer cell signal transduction, regulating the cell cycle, and modulating the expression of apoptosis-related proteins. Guo’s research investigated the mechanisms by which cordycepin inhibits HepG2 and Huh7 liver cancer cells using migration, invasion, and chemotaxis assays. They found that the invasion and migration rates of cells treated with 10 µM cordycepin were significantly lower than those of the control group, with a concomitant downregulation of CXCR4 expression. Consequently, the migration and invasion abilities of liver cancer cells were impaired, indicating a significant inhibitory effect of cordycepin on liver cancer. Furthermore, the inhibitory effect of cordycepin was markedly enhanced when used in combination with JSH-23, suggesting that cordycepin may have potential to prevent liver cancer metastasis when combined with other therapeutic compounds (62). Li et al. treated HepG2 cells with various concentrations of cordycepin and doxorubicin, either alone or in combination, for 48 hours. Both cordycepin and doxorubicin downregulated CCL2 expression in a dose-dependent manner, effectively inhibiting HCC cell proliferation and migration, and inducing apoptosis. The combined treatment showed more pronounced effects (85).

#### 3.1.2 Sinomenine

Sinomenine is an alkaloid compound primarily extracted from the roots and stems of the Menispermaceae plant, *Sabia japonica* Maxim., commonly used in its hydrochloride salt form (86). It exhibits a broad range of pharmacological activities, including antitumor, anti-inflammatory, neuroprotective, and immunosuppressive properties, making it highly effective against HCC (87). Shen et al. investigated the inhibitory effects of sinomenine hydrochloride on the growth, invasion, and metastasis of mouse liver cancer cells (Hepa1-6). They analyzed its impact on the expression of apoptosis-related and metastasis/invasion-related genes at the mRNA level. The study found that sinomenine hydrochloride inhibits the CXCL12-CXCR4 and CCL21-CCR7 axes in HCC cells, thereby suppressing HCC cell growth and invasion while promoting apoptosis (63).

#### 3.1.3 Conophylline

Conophylline (CnP), a vinca alkaloid derived from the tropical plant *Ervatamia microphylla* Pit., has shown promising therapeutic potential for liver disease (88). Utilizing a primary CAFs model established from surgically resected HCC tissues, it was revealed that CnP effectively inhibits the activation of rat HSCs. Further investigations demonstrated that CnP primarily exerts its anti-HCC effects by suppressing the activation of CAFs and reducing the production of pro-tumorigenic factors, particularly interleukin-6 (IL-6), interleukin-8 (IL-8), CCL2, angiopoietin, and osteopontin (OPN) (64).

#### 3.1.4 Isorhynchophylline

Isorhynchophylline (Rhy), a major oxindole alkaloid isolated from *Uncaria rhynchophylla* (Miq.) Miq. ex Havil., has demonstrated notable anticancer properties. Lee et al. employed HepG2 cells to elucidate the detailed mechanisms underlying Rhy’s anticancer effects. Utilizing Annexin V assay, scratch assay, and Transwell assay, their findings revealed that Rhy inhibits HepG2 cell migration, invasion, and the constitutive expression of CXCR4. Furthermore, the study showed that Rhy modulates multiple cellular signaling pathways by inhibiting the phosphorylation of p38, ERK, JNK, CREB, c-Jun, Akt, and STAT3, while enhancing the phosphorylation of p53 at the Ser15 residue. These actions collectively induce apoptosis and exert antimetastatic effects in HepG2 cells (65).

### 3.2 Phenols

Phenolic compounds, a class of organic molecules featuring one or more hydroxyl groups (-OH) directly attached to an aromatic ring, are abundant in various natural products, including *Scutellaria baicalensis* Georgi, *Salvia miltiorrhiza* Bunge, and catechins. The CCL2/CCR2 axis has been implicated in HCC by promoting the recruitment of monocytes/macrophages. Deepak et al. conducted a systematic investigation into the inhibitory mechanisms of epigallocatechin-3-gallate (EGCG) and gallocatechin gallate (GCG) in HCC. Their findings demonstrated that both EGCG and GCG inhibit CCL2 chemokine-mediated monocyte recruitment. Notably, EGCG reduces CCL2-induced macrophage migration, whereas GCG functions as an effective protein-protein interaction (PPI) inhibitor, regulating CCL2-directed leukocyte recruitment to ameliorate inflammation and immune dysregulation (66).

### 3.3 Polysaccharide

Polysaccharides are polymeric carbohydrates consisting of ten or more monosaccharide units linked by glycosidic bonds. They are prevalent in animals, plants, microorganisms, and other organisms. Owing to their low toxicity and minimal side effects, polysaccharides have found extensive applications in the food and pharmaceutical industries (89). Research has demonstrated their antitumor effects through mechanisms including the regulation of the CXCR4/CXCL12 axis, downregulation of CXCL12 expression, and reduction in the production of eosinophil chemotactic factors (90).

#### 3.3.1 Ganoderma resinaceum polysaccharide

*Ganoderma resinaceum* polysaccharide-rich fractions (GRP) exhibits chemopreventive properties by inhibiting cell proliferation, promoting liver structural restoration, enhancing antioxidant enzymes, and modulating cytokine/chemokine levels (67). Data from the treatment groups reveal that GRP II effectively reduces immune cell infiltration into subcutaneous tissues, suppresses the expression of pro-inflammatory cytokines Interferon-γ (IFN-γ), tumor necrosis factor-α (TNF-α) and chemokines (eosinophil chemotactic factor, fractalkine), and increases levels of anti-inflammatory cytokines (IL-10 and IL-12p70) in Wistar rats with N-nitrosodiethylamine-induced HCC, highlighting its potent *in vivo* anti-inflammatory activity (67).

#### 3.3.2 Fucoidan

Fucoidan, also known as brown algae polysaccharide, is a natural active polysaccharide uniquely found in brown algae and containing sulfate groups (91). It primarily resides in the cell wall matrix, intercellular spaces, and secreted mucilage of seaweeds such as kelp, wakame, giant kelp, and bladderwrack. Fucoidan exhibits essential functions including antimicrobial, moisturizing, and radioprotective effects on algae themselves (92). Recent research has extensively documented multiple physiological benefits of fucoidan, including enhancement of gastrointestinal health, anti-tumor properties, treatment of chronic kidney failure, immune modulation, and anticoagulant activity. Studies have demonstrated that fucoidan modulates the CXCL12/CXCR4 axis, exerting a dose-dependent inhibitory effect on Huh7 liver cancer cells by reducing CXCL12 expression (68). Fucosylated AAL-polysaccharides and LCA-glycoproteins similarly significantly decrease IL-8-induced migration of HCC cells. This effect may be attributed to their capacity to bind IL-8 receptors, particularly CXCR2, which likely retains sugar residues linked with Fuc α1-2 and/or Galβ1-4 (Fucα1-3) GlcNAc. This interaction competitively inhibits IL-8 binding to CXCR2, thereby suppressing the migration of human liver cancer cells (69).

### 3.4 Flavonoids

Flavonoids, secondary metabolites naturally found in plants, demonstrate diverse biological activities (93). In anticancer research, several flavonoids have been identified to modulate the expression or activity of chemokines and their receptors to exert their effects.

#### 3.4.1 Breviscapine

Breviscapine, a flavonoid isolated from the traditional Chinese herb *Erigeron breviscapus* (Vant.) Hand.-Mazz., is renowned for its diverse pharmacological effects, including anti-inflammatory, antioxidant, anti-apoptotic, vasodilatory, antiplatelet, and anticoagulant properties. Recent studies have demonstrated that breviscapine protects against CCl4-induced liver injury by reducing the secretion of pro-inflammatory cytokines and oxidative stress. Specifically, breviscapine attenuates the secretion of TNF-α, IL-6, IL-1β, and the chemokine monocytechemoattractantprotein-1 (MCP-1) in serum, as well as their expression in liver tissues (70).

#### 3.4.2 Icaritin

Icaritin (ICT), an active flavonoid compound derived from *Epimedium* Linn., has demonstrated clinical efficacy in extending the survival of HCC patients through immune modulation (94). Several studies have shown that ICT induces autophagy and apoptosis in cancer cells while enhancing the anti-tumor effects of doxorubicin. However, the precise mechanisms underlying these effects remain to be elucidated. Yu et al. first reported that ICT inhibits the proliferation of Hepa1-6 and Huh7 liver cancer cells by promoting caspase-mediated mitochondrial apoptosis. Furthermore, they demonstrated that the combination of ICT and doxorubicin synergistically reduces the immunosuppressive functions of MDSCs, Treg cells, and M2 macrophages, leading to decreased release of immunosuppressive cytokines, including chemokine CCL2, transforming growth factor β (TGF-β), IL-4, IL-6, and IL-10 (95). Additional research has revealed that ICT significantly blocks the immunosuppressive activity of bone marrow cells, modulates immunosuppressive MDSC cells and inflammation-associated cytokines and chemokines, and inhibits the expression of programmed death ligand-1 (PD-L1), thereby enhancing the efficacy of immunotherapy and anti-cancer treatments (71).

#### 3.4.3 Flavokawain A

Xiao’s research isolated a chalcone compound, flavokawain A (FKA), from *Chloranthus henryi* Hemsl. Utilizing SMMC-7721, Huh7, PANC-1, HepG2, HeLa, and Hep3B cell lines, they conducted Transwell invasion assays to investigate cell invasiveness. Their findings indicate that FKA inhibits the migration, invasion, VM formation, and EMT progression of HCC cells by targeting CXCL12, thereby suppressing the PI3K/Akt/HIF-1α/Twist1 pathway (72).

### 3.5 Plumbagin

Zhong et al. utilized SDF-1 to induce proliferation, invasion, and growth factor activation in the HCC cell line SMMC-7721, revealing that SDF-1 enhances the secretion of angiogenic factors IL-8 and VEGF. Plumbagin (PL) was found to significantly inhibit SDF-1-induced angiogenesis in co-cultured SMMC-7721 and HUVECs, and it downregulated the mRNA expression of CXCR4 and CXCR7, thereby suppressing angiogenesis in HCC (73).

### 3.6 Tannic

*Terminalia bellirica* (Gaertn.) Roxb. (TB-TF) tannin extract promotes T cell infiltration mediated by the chemokines CCL5 and CXCL10. Chemokine CCL5 and its receptor CCR5, as well as CXCL9, CXCL10, and their receptor CXCR3, are involved in the recruitment of CD8+ T cells within the TME (96). Immunohistochemical (IHC) staining with CD68, a pan-macrophage marker, was employed to evaluate macrophage recruitment. The results showed that TB-TF treatment significantly increased the number of tumor-infiltrating macrophages in Hepa 1-6 orthotopic mice and markedly enhanced macrophage-mediated recruitment of CD8+T cells. Restoring the ability of tumor-trained macrophages to recruit CD8+T cells inhibited HCC growth and reversed tumor-conditioned media-induced M2 polarization of macrophages. However, the underlying mechanisms by which TB-TF reverses this tumor cell suppression remain unclear and warrant further investigation (74).

### 3.7 Terpenes

Terpenoids are a class of natural products commonly found in plants, known for their diverse biological activities, including anti-inflammatory, antibacterial, and antitumor properties. In the realm of antitumor research, the antitumor potential of terpenoids has been extensively investigated. Current studies suggest that cantharidin and triptolide may exert their antitumor effects by modulating chemokines and their receptor signaling pathways, thereby influencing the activities of immune cells and tumor cells.

#### 3.7.1 Cantharidin

Cantharidin (CTD), the primary component of an anticancer drug derived from *Mylabris phalerata* Pallas, has shown significant antitumor activity in various cancers, particularly HCC (97). *In vivo* treatment with CTD resulted in increased expression of CXC and CCL chemokines involved in the immune response. Flow cytometry analysis of mouse blood indicated an elevated proportion of CD4+/CD8+ T cells and B cells, while the proportion of T lymphocytes decreased. These findings suggest that CTD may inhibit HCC progression by modulating chemokines that regulate immune cell trafficking and immune signaling responses (75).

#### 3.7.2 Celastrol

Celastrol, a compound extracted from the traditional Chinese herb *Tripterygium wilfordii* Hook. f., exhibits notable antitumor activity. Its antitumor mechanisms primarily include inhibiting tumor cell proliferation, promoting tumor cell apoptosis, suppressing tumor cell migration, and inhibiting tumor angiogenesis. Chan et al. discovered that triptolide dose-dependently reduces CXCR4 expression by inhibiting the CXCR4-mediated pathway. This inhibition also downregulated downstream pathways, including PI3K and Akt. Their experimental results demonstrated that triptolide significantly inhibits the proliferation and migration of HCC cells and induces apoptosis by targeting CXCR4-related signaling pathways (76).

### 3.8 Steroids

Current research indicates that steroid compounds have potential therapeutic effects in HCC treatment through modulation of chemokines (98). Yang et al. observed elevated expression of APOBEC3F in HCC tumors compared to adjacent tissues based on proliferation and migration experiments, suggesting it as a potential tumor protein influencing HCC invasiveness. Further confirmation through APOBEC3F siRNA experiments showed that siAPOBEC3F reduced the expression levels of intestinal immune network IgA generation pathway proteins, including CCR9, CCR10, CXCR4, and pIgR. Subsequently, in SK-Hep1 and Bel-7404 cell lines, the effects of bufalin on cell proliferation and migration were assessed via CCK-8 assay, wound healing assay, and transwell assay. Bufalin was found to inhibit the expression of CCR9, CCR10, CXCR4, and pIgR proteins, thereby suppressing IgA production and impeding cancer cell proliferation and migration (77).

### 3.9 Phenylpropanoids

*Schisandra chinensis* (Turcz.) Baill is a perennial deciduous woody vine in the Magnoliaceae family (99). A recent study by Jiang et al. revealed that the combination of the Schisandra chinensis lignans and acteoside effectively suppressed the expression of CCL20. This is noteworthy because the CCL20-CCR6 axis is implicated in promoting invasion and metastasis of hepatocellular carcinoma cells by influencing regulatory T cells within the tumor microenvironment. The study suggests that the Schisandra chinensis lignans and acteoside may counteract CCL20-induced epithelial-mesenchymal transition (EMT), invasion, and migration of hepatocellular carcinoma cells via the ERK1/2 pathway, while also promoting apoptosi (78). Osthole is a natural coumarin compound extracted from the dried mature fruits of the Umbelliferae plant *Cnidium monnieri*. Liu and colleagues utilized an SD rat model of thioacetamide (TAA)-induced liver fibrosis to demonstrate osthol’s significant reduction in fibrosis-related gene expression induced by TAA. Analysis of various extracellular matrix (ECM) formation mediators via cytokine arrays revealed that osthol treatment markedly decreases levels of ICAM-1, CD62L, VEGF, and the chemokine CX3CL1. These findings highlight osthol’s potent inhibitory effects on the inflammatory response associated with liver fibrosis (79).

### 3.10 Other natural product extracts

Natural product extracts have shown promising potential in regulating chemokine expression for the treatment of HCC. Specific natural product extracts have been identified for their ability to modulate chemokine expression, thereby impeding the progression of HCC. These extracts exert their effects by targeting specific signaling pathways or molecular targets involved in the generation and secretion of chemokines, thus modifying the tumor microenvironment to suppress tumor growth and metastasis. For instance, *Oldenlandia diffusa* (Willd.) Roxb. (OD) extract, a natural product, inhibits HCC metastasis by downregulating crucial migration-related chemokine receptors such as CXCR1, CXCR2, and CXCR4. Scratch wound healing assays conducted with Huh7 and HepG2 cells have demonstrated that OD inhibits the migration of liver cancer cells through the inhibition of OA and UA migration receptors. Moreover, OD enhances the expression of the apoptosis-related enzyme caspase-3, thereby suppressing the growth of liver cancer cells (80). Trichosanthin (TCS) is a single-chain ribosome-inactivating protein extracted from the rhizomes of the natural product *Trichosanthes* Linn (100). Wang et al. explored the *in vitro* and *in vivo* anticancer effects of TCS in HCC by administering it to HCC cell cultures and xenograft models in BALB/c mice with intact immune systems. They investigated how TCS modulates T cell recruitment in host anti-HCC immune responses. Treatment with varying concentrations of TCS demonstrated dose-dependent inhibition of HCC cell line and xenograft tumor growth. Further investigations showed that TCS upregulates the expression of chemokines CCL2, CCL17, and CCL22, facilitating the accumulation of CD8+ T cells in HCC tissues (81). Additionally, TCS-induced GrzB secretion by T cells, transported via M6PR into HCC cells, promotes cell apoptosis (101). These findings suggest that TCS enhances T cell-mediated immunity in anti-tumor immunotherapy by potentially augmenting chemokine secretion and facilitating GrzB entry into HCC cell (81). Cisplatin (CDDP), a platinum-based anticancer drug, markedly upregulates the expression of chemokine CKLF1 in HepG2 cells. Functional assays reveal that CKLF1 potentially enhances metastasis in HCC. Kanglaite (KLT), derived from *Coix lacryma-jobi* L.var.mayuen(Roman.) Stapf seed, mitigates CDDP-induced CKLF1-mediated NF-κB pathway activation, thereby synergizing with CDDP in the combined therapy of HCC (102). Yang et al. established a subcutaneous tumor model in mice and found that treatment with AAE significantly reduced tumor size and weight. Immunohistochemical staining revealed that AAE intervention significantly decreased the proliferative capacity of tumor cells and increased the expression of multiple chemokines, including CCL2, CCL5, and CCL10. Notably, CCL2 was the most upregulated chemokine following AAE treatment, significantly enhancing NK cell infiltration into tumors. The knockdown of CCL2 via viral transfection attenuated AAE’s effects on NK cell infiltration and tumor growth inhibition, further validating these findings. The authors conducted *in vitro* studies using HuH7, HepG2, and Hepa1-6 cell lines, elucidating that the upregulation of CCL2 expression is mediated through JNK activation. Viral transfection-mediated knockdown of CCL2 diminished AAE’s effects on NK cell infiltration and tumor growth inhibition (29). Cisplatin (CDDP) is a widely employed chemotherapeutic agent for treating hepatocellular carcinoma. While elevating its concentration enhances cancer cell apoptosis rates, it concurrently poses risks of toxicity and resistance, thereby constraining its clinical utility (103). Kanglaite (KLT) is a biologically active compound derived from Coix lacryma-Jobi, recognized as a biphasic broad-spectrum anticancer agent (104). KLT augmented the antitumor efficacy of CDDP in HepG2 cells. While CDDP notably upregulated the chemokine-like factor 1 (CKLF1)-mediated NF-κB pathway in HepG2 cells, KLT effectively suppressed CDDP-induced NF-κB activation. This synergistic effect possibly stems from its ability to modulate inflammation and combat chemotherapy resistance (102). The mechanism underlying the combined therapy of KLT and CDDP against hepatocellular carcinoma remains unclear, necessitating comprehensive studies. Nevertheless, these findings advance our understanding of the pharmacological interactions between traditional Chinese and Western medicines, highlighting their potential in treating hepatocellular carcinoma.

## 4 Role of botanical drugs remedies in hepatocellular carcinoma

Botanical drugs remedies are a pivotal aspect of traditional medicine, distinguished by their multi-component and multi-target characteristics (105). These formulations regulate the body’s internal environment and enhance immune function, thereby achieving therapeutic effects (106). Recent studies have increasingly highlighted the efficacy of natural product formulations in treating HCC (**Table 2**).

**Table 2:** Mechanism of botanical drugs remedies in the treatment rough the chemokines.

Botanical drugs remedies	Chemokines or chemokine receptors	Machine	References
Astragalus mongholicus Bunge—Curcuma aromatica Salisb	CXCL8/CXCR2	Downregulate the CXCL8/CXCR2 axis in colorectal cancer, inhibit the PI3K/Akt/mTOR pathway, suppress the EMT process, and mitigate liver metastasis	(107)
Biejiajian Pill	CCL5	Induce the expression of CCL5 to promote the infiltration of peripheral blood CD8+ T cells into tumor tissues via the CCL5 pathway	(108)
Gehua Jiecheng Decoction	CCL2	Counteract the immune-suppressive effects of the hepatocellular carcinoma microenvironment, exert anti-inflammatory and anti-angiogenic activities, downregulate the proportions of Tregs, TAMs, and MDSCs, upregulate the proportions of CD8 T cells and functional CD8 T cells, inhibit the expression of IL-6, IL-10, TNF-α, and CCL-2, and reduce the expression of angiogenesis-related molecules CD31 and VEGF	(109)
Shipi-Xiaoji recipe	ACKR3	Inhibition of HEPG2 proliferation and invasion by dose-dependent inhibition of RGCC and ACKR3 protein expression	(110)
Kuan-Sin-Yin	CCL2	Downregulate CCL2, CEACAM1, and PIK3R3 to inhibit the migration of hepatocellular carcinoma cells	(111)
Tianhuang formula	CCL2-CCR2	Inhibit the CCL2-CCR2 axis and MAPK/NF-κB activation to alleviate macrophage infiltration and activation of hepatic stellate cells (HSCs)	(112)
Dahuang Zhechong Pill	CCL2	Inhibit the CCL2-mediated M2-skewing paradigm to improve the pro-fibrotic microenvironment and suppress liver metastasis of CRC	(113)
Wan-Nian-Qing prescription	CCL28	Modulate Nrf2 and its downstream proteins to inhibit oxidative stress, counteract ROS generation and accumulation, enhance SOD activity, regulate IL-10 levels, activate NK cell secretion of CCL28 upon IL-2 binding, and augment targeted cytotoxicity against tumor cells	(114)
Qizhu Anticancer Prescription	CXCL14	The upregulation of p21 expression and secretion of PASP factor in tumor cells lead to irreversible cell cycle arrest under cellular stress.	(115)
Chanling Gao	CXCR4	Inhibit the HIF1α/SDF1α-CXCR4/PI3K-AKT signaling pathway to reduce vascular endothelial growth factor synthesis and release, decrease type IV collagen degradation, and suppress tumor growth and liver metastasis in nude mice	(116)
JC-001	CCL1/CCL17	Inhibit Th17 immunity, leading to elevated levels of IL-1, IL-6, IL-10, and IL-12 p70. IL-12 facilitates the differentiation of undifferentiated CD4+ T cells into Th1 cells, which produce CCL1 and CCL17, directly promoting cytotoxicity and proliferation of CD8+ T cells, and inducing T cells and NK cells to secrete TNF-α and IFN-γ.	(117)

The use of *Astragalus mongholicus* Bunge—*Curcuma aromatica* Salisb. (AC) is a classic pairing in Chinese medicine for the treatment of HCC disease (118). AC modulates the CXCL8/CXCR2 chemokine axis and inhibits the PI3K/Akt/mTOR pathway in colorectal cancer cells and orthotopic mouse models, thereby exerting anti-metastatic effects on HCC (107). Biejiajian Pill (BJJP), originally documented in the “Jin Gui Yao Lue”. Research indicates that BJJP inhibits tumor cell growth in an immune-dependent manner, modulating CCL5 expression and promoting CD8+ T cell infiltration into HCC tumors in H22 tumor-bearing mice (108). Cheng et al. demonstrated that Gehua Jiecheng Decoction (GHJCD) has therapeutic effects on diethylnitrosamine (DEN)-induced HCC in mice. Their findings elucidate that GHJCD counteracts the immunosuppressive tumor microenvironment of HCC by downregulating the proportions of Tregs, TAMs, and MDSCs (109). Zhang et al. utilized bioinformatics and network pharmacology to identify that the regulator of cell cycle gene (RGCC) and atypical chemokine receptor 3 (ACKR3) are implicated in the progression from non-alcoholic liver disease to hepatocellular carcinoma and impact the prognosis of hepatocellular carcinoma. They discovered that the Shipi-Xiaoji recipe (SPXJF) effectively inhibited the proliferation and invasion of HEPG2 cells in a dose-dependent manner. Furthermore, SPXJF was observed to dose-dependently suppress the expression of RGCC and ACKR3 proteins, highlighting its potential therapeutic relevance (110). The Chinese herbal decoction Kuan-Sin-Yin (KSY) reduces the expression of CCL2, which is associated with cell migration, and downregulates the mRNA levels of phosphoinositide-3-kinase regulatory subunit 3 (PIK3R3) and CEA cell adhesion molecule 1 (CEACAM1), thereby inhibiting the migration and invasion of HCC cells (111). Wang et al. explored the mechanisms by which Jiedu Huayu granules prevent liver injury in a rat model induced by D-galactosamine and lipopolysaccharide, demonstrating that these granules mitigate liver damage through T cell-mediated suppression of inflammation (119). Tianhuang formula (THF) downregulates the macrophage marker CD68 and improves liver injury, inflammation, and fibrosis by inhibiting the CCL2-CCR2 axis and its downstream MAPK/NF-κB signaling pathways (112). Dahuang Zhechong Pill (DZP) reduces the expression of CCL2 and its receptor CCR2 in the liver, decreasing M2 macrophage polarization. By remodeling the hepatic microenvironment, it inhibits colorectal cancer liver metastasis, providing strong evidence for the role of traditional Chinese medicine in reshaping the premetastatic niche and preventing liver metastasis (113). The Wan-Nian-Qing prescription (WNQP), a traditional Chinese medicine formulation containing Ornithogalum caudatum, has shown promising immunomodulatory effects. Antibody chip screening in immunosuppressed BALB/c mice revealed that WNQP modulates serum levels of interleukins and chemokines, leading to IL-2 activation of NK cells and subsequent secretion of CCL28. This mechanism enhances the targeted cytotoxicity against tumor cells, forming an immunoregulatory network that inhibits tumor growth and regulates tumor progression (114). When it comes to treating liver diseases, the Qizhu Anticancer Prescription (QZACP) has shown promise as a therapeutic approach (120). In DEN-induced hepatocellular carcinoma mice, QZACP significantly reduced both the number and size of intrahepatic nodules. It effectively mobilized NK and CD8+ T cells to combat tumor invasion and stimulated the release of interferon-γ. Furthermore, QZACP induced irreversible cell cycle arrest in stressed tumor cells by up-regulating the expression of p21 and promoting the secretion of PASP factors (decorin, CXCL14, and Wnt family member 2) within tumor cells after recruiting NK and CD8+ T cells (115). Yang et al. demonstrated that the binding of SDF-1 to CXCR4 activates downstream signaling pathways that regulate tumor cell proliferation, adhesion, and migration. The traditional Chinese medicine formulation Chanling Gao (CLG) was found to inhibit the expression of SDF-1α and CXCR4 in the liver. Results indicate that CLG effectively suppresses the growth and liver metastasis of colorectal cancer xenografts in nude mice, potentially through the HIF-1α/SDF-1α-CXCR4/PI3K-Akt signaling pathway (116). JC-001 is a traditional Chinese medicine formulation used for the treatment of liver diseases. Clinical studies have demonstrated that JC-001 inhibits Hepa 1-6 tumors in immunocompetent models through immunomodulation. It increases TNF-αlevels in the tumor microenvironment, thereby enhancing the inflammatory response. Additionally, JC-001 induces an elevation in IL-12p70 levels within the tumor microenvironment. IL-12 enhances the cytotoxicity, survival, and proliferation of CD8+ T cells by producing CCL1 and CCL17. It also aids in the differentiation of naive CD4+ T cells into Th1 cells, promoting the secretion of TNF-α and IFN-γ by T cells and NK cells (117). Botanical drugs remedies have shown efficacy and advantages in the treatment of HCC, involving multiple mechanisms. However, current research on their use for HCC remains in the early stages, and further in-depth investigation and validation of their mechanisms of action and efficacy are required.

## 5 Discussion and prospect

Currently, there are numerous treatments for HCC, including surgical resection, liver transplantation, radio frequencyablation (RFA), transcatheter arterial chemoembolization (TACE), targeted therapy, and immunotherapy (121). Due to the subtle symptoms of early-stage HCC, early detection is often challenging, making surgical eradication difficult at diagnosis. Surgical interventions, particularly liver transplantation, are theoretically effective but limited in practice by donor shortages and eligibility criteria (122). Liver resection also has drawbacks, including lower recurrence-free survival rates and high risks for patients with advanced cancer and cirrhosis (123). Radio frequencyablation techniques are minimally invasive, safe, simple, and relatively inexpensive, but they require repeated treatments to be effective and are limited by tumor size and location (124). TACE is an interventional procedure that delivers chemotherapeutic drugs directly to liver tumors via the hepatic artery, effectively targeting the tumor tissue (125). Although TACE can improve survival rates in intermediate-stage patients, the complex arterial supply of HCC means that tumors may continue to grow via collateral circulation even after main vessel embolization, leading to postoperative recurrence (126). Chemotherapy for HCC also carries risks. For instance, sorafenib, a multi-kinase inhibitor, can block up to 40 kinases, extending the survival time of patients with advanced HCC from 7.9 months to 10.7 months (127). However, its adverse effects, such as hand-foot skin reactions, diarrhea, liver function abnormalities, hypertension, and rash, as well as the potential for drug resistance within six months of treatment initiation, limit its clinical efficacy in treating HCC (128).

HCC resides within a complex immune microenvironment, where the TME often exerts functional suppression on immune cells, thereby playing a pivotal role in cancer progression (129). The genesis and dynamic alterations of this TME encompass diverse cell types and intricate signaling pathways, reminiscent of the multi-faceted and bidirectional immune modulation emphasized in traditional Chinese medicine (130, 131). Natural products leverage their advantages such as comprehensive regulation and minimal toxicity, complementing conventional therapies by ameliorating patient symptoms, bolstering immune function, and enhancing overall quality of life during HCC treatment. Natural products, enriched with multiple active constituents, confers diverse mechanisms of action and targets, thus influencing the pathological processes of HCC through multifaceted approaches concurrently (132). Based on current research, we are increasingly clarifying the mechanisms through which natural products exert therapeutic effects in HCC by modulating chemokine function. Certain active ingredients of natural products inhibit chemokine production, thereby reducing immune cell aggregation and inflammation. Conversely, other active ingredients can stimulate chemokine expression, enhancing immune system function and suppressing tumor growth and metastasis (133). Chemokines, a significant research focus in biomedicine, play recognized roles in immune and inflammation-related diseases (134). Chemokine-based therapies in immunotherapy generally fall into two categories: targeting pro-tumor chemokines and increasing anti-tumor chemokine levels. Both can be used as standalone therapies or in combination with other treatment strategies. There have been successful clinical applications of chemokine therapies, such as the CXCR4 antagonist AMD3100 for treating relapsed or refractory acute myeloid leukemia (AML) and the CCL2 inhibitor CNTO 888 for metastatic prostate cancer (135). These developments provide new treatment options for cancer patients and lay a solid foundation for further advancement in chemokine therapies. Furthermore, data suggest that combining natural products with chemotherapy drugs significantly improves chemotherapy sensitivity, enhances tumor suppression effects, and notably ameliorates cancer-related fatigue, bone marrow suppression, and other adverse reactions (136). Natural products typically exhibit multi-target and multi-pathway characteristics in cancer treatment, effectively impeding HCC development by modulating various physiological processes (137). However, despite some successes, natural products in HCC treatment face challenges. The development and progression of HCC involve abnormalities in multiple genes and pathways, making it difficult for a single drug to address all aspects. Additionally, the complex composition and unclear mechanisms of natural products limit their application in HCC treatment. Chemokine therapies still face many challenges and opportunities. The complexity of chemokines and their receptors makes selecting and validating therapeutic targets particularly difficult. Nonetheless, with the continuous development and innovation of biotechnology, techniques like gene editing and the combined application of immunotherapy and chemokine therapies could allow more precise regulation of chemokine expression and function, achieving targeted disease treatment.

**Figure 2:**
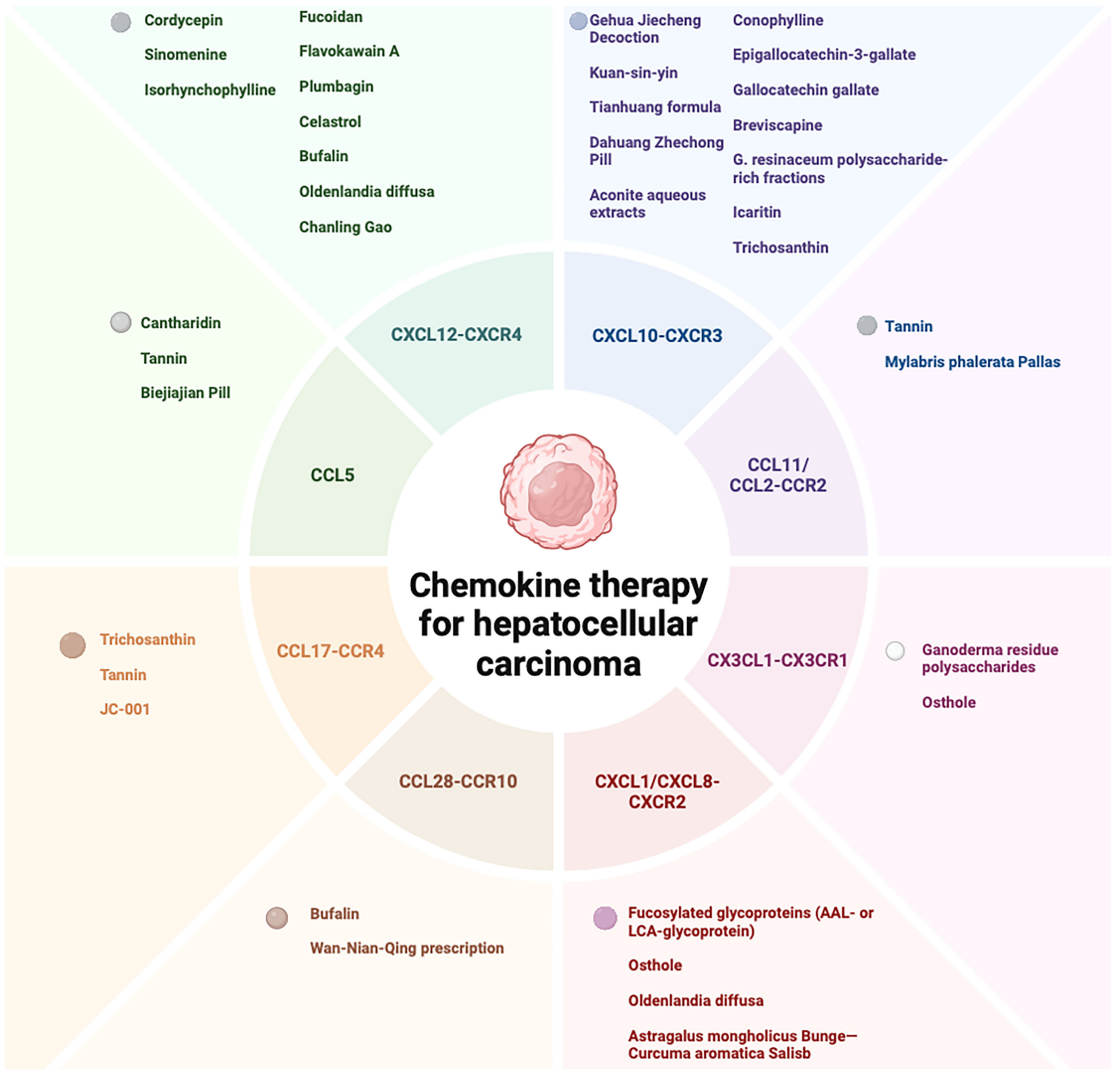
Therapeutic potential of natural products and its extracts, and formulations in HCC treatment via chemokine modulation.

In summary, our comprehensive investigation into the role of chemokines within the immune microenvironment of HCC offers a novel perspective on understanding the disease’s pathogenesis (138). Concurrently, the exploration of natural product-derived active ingredients targeting chemokines in HCC treatment presents promising new therapeutic avenues (**Figure 2**). However, while natural products offer distinct advantages in HCC treatment, they also come with certain limitations (139). Thus, it is crucial to elucidate the precise mechanisms through which natural products modulate chemokines to enhance therapeutic outcomes in HCC. This effort aims to provide enhanced theoretical support and practical guidance for precision medicine in HCC treatment. As our understanding of HCC pathogenesis advances, future research may uncover additional therapeutic targets involving chemokines, potentially leading to more effective treatment strategies for HCC patients.
